# Multistrain Probiotics and Telomere Length in Type 2 Diabetes: A 24-Week Randomized Controlled Trial

**DOI:** 10.3390/life15020311

**Published:** 2025-02-17

**Authors:** Venkata Chaithanya, Janardanan Kumar, Kakithakara Vajravelu Leela, Habeeb Ali Baig, Mohamed Soliman, Awwad Alenezy, Naglaa M. Shalaby

**Affiliations:** 1 Department of Microbiology, SRM Medical College Hospital and Research Centre, SRMIST, Kattankulathur, Chengalpattu 603203, Tamil Nadu, India; vd6943@srmist.edu.in (V.C.); leelav@srmist.edu.in (K.V.L.); 2 Department of General Medicine, SRM Medical College Hospital and Research Centre, SRMIST, Kattankulathur, Chengalpattu 603203, Tamil Nadu, India; 3 Department of Microbiology, Faculty of Medicine, Northern Border University, Arar 91431, Saudi Arabia; docbaig@yahoo.com (H.A.B.); mohamed.sherif@nbu.edu.sa (M.S.); nagla.shalaby@nbu.edu.sa (N.M.S.); 4 Department of Family and Community Medicine, Faculty of Medicine, Northern Border University, Arar 91431, Saudi Arabia; dr.awwad@hotmail.com

**Keywords:** type 2 diabetes, probiotics, telomere length, HbA1c, hs-CRP

## Abstract

Background: This 24-week randomized controlled trial aimed to evaluate the impact of multistrain probiotic supplementation on telomere length in patients with type 2 diabetes (T2DM). The study also assessed secondary outcomes including high-sensitivity C-reactive protein (hs-CRP) and glycated hemoglobin (HbA1c). Methods: A total of 124 participants with T2DM were randomly assigned to either a probiotic group (*n* = 62) or a placebo group (*n* = 62). Participants in the probiotic group consumed a supplement containing fourteen live microbial strains, including *Lactobacillus plantarum*, *L. fermentum*, *L. acidophilus*, *L. casei*, *L. rhamnosus*, *L. reuteri*, *L. salivarius*, *L. paracasei*, *L. gasseri*, *Bifidobacterium bifidum*, *B. lactis*, *B. breve*, *Streptococcus thermophilus*, and *Saccharomyces boulardii*, with each strain providing 2.148 billion CFUs per capsule, totaling 30 billion CFUs. The placebo group received vitamin B12 capsules without probiotics. The primary outcome was telomere length, and secondary outcomes included hs-CRP and HbA1c levels. Data were analyzed using intention-to-treat (ITT) and per-protocol (PP) methods. Results: The probiotic group exhibited a statistically significant decrease in telomere shortening compared to the placebo group (*p* < 0.001). The hs-CRP levels decreased more significantly in the probiotic group (*p* < 0.001), suggesting potential anti-inflammatory effects. The HbA1c levels improved in the probiotic group, with a reduction of 0.44% (*p* = 0.004). An age-stratified analysis revealed more substantial improvements in the 30–49 years cohort, which showed greater reductions in telomere shortening, inflammatory markers, and metabolic indicators compared to the 50–69 years group. Conclusions: Multistrain probiotic supplementation shows potential benefits in reducing telomere shortening and improving glycemic control. However, further long-term studies are needed to understand the underlying mechanisms and therapeutic implications of probiotics in T2DM. Trial registration: This trial was registered at the Clinical Trials Registry-India (CTRI/2023/07/055647).

## 1. Introduction

Telomere shortening has been found as a primary mechanism causing early cell senescence, which is increasingly becoming recognized as a cause and consequence of type 2 diabetes mellitus (T2DM) and its complications. Telomeres are unique DNA-protein complexes found at the ends of chromosomes that preserve genomic integrity and protect them from degradation [1,2]. Telomerase, the enzyme that prevents telomere shortening, is made up of telomere reverse transcriptase (TERT) and a telomerase RNA component [3]. Therefore, telomere length is commonly used as a biological marker for cell aging. Aging and genomic instability have been connected to telomeric DNA’s susceptibility to oxidative stress, which can cause single-strand breaks and telomere degradation [4]. Elevated oxidative stress makes telomere shortening more prevalent in diseases like prediabetes and metabolic syndrome [5]. Hyperglycemia accelerates telomere shortening by increasing the production of reactive oxygen species (ROS), which initiate cellular damage. The persistent accumulation of ROS and their by-products leads to oxidative stress. The free radical theory of aging suggests that the buildup of oxidative stress over time contributes to the aging process of an organism [6]. Given this relationship, telomere shortening may be a biomarker of cellular aging and cumulative damage in people with type 2 diabetes [7].

Insulin resistance, poor glucose metabolism, and several consequences, including cardiovascular disease, renal dysfunction, and neuropathy, are hallmarks of type 2 diabetes, a condition that is becoming a major global health concern [8]. Complementary therapies like probiotic supplements are gaining popularity, but traditional treatments like medication, insulin therapy, and lifestyle changes continue to be the mainstay of managing type 2 diabetes. The ability of probiotics, particularly multistrain formulations, to alter gut microbiota and lower systemic inflammation—two factors that are critical to the pathogenesis of type 2 diabetes—is drawing more attention [9]. Studies have indicated that probiotics may affect a number of biomarkers linked to the advancement of type 2 diabetes, such as glycated hemoglobin A1c (HbA1c) and high-sensitivity C-reactive protein (hs-CRP) [10].

Probiotics have been shown to reduce hs-CRP levels by modulating immune responses and decreasing gut-derived inflammation, offering potential benefits in reducing the chronic inflammation associated with T2DM. HbA1c, a long-term indicator of blood glucose control, is another critical marker in T2DM management. Poor glycemic control, reflected in high HbA1c levels, is associated with an increased risk of complications. Probiotics may enhance insulin sensitivity, regulate glucose metabolism, and influence gut microbiota composition, potentially leading to improved glycemic control and reduced HbA1c levels [11]. Probiotics may slow down telomere attrition and assist in maintaining telomere length at the cellular level [12].

Though preliminary research indicates that probiotics may have positive benefits on these biomarkers, the evidence is still unclear, especially when it comes to multistrain probiotic administration in T2DM patients. The rationale for selecting probiotic strains, such as *Lactobacillus acidophilus*, *Lactobacillus casei*, and *Lactobacillus plantarum*, is based on their ability to improve glucose metabolism and lower inflammatory markers in metabolic disorders like diabetes. While studies on multistrain probiotics in diabetes are limited, their potential effects on telomere length remain unexplored and warrant further investigation. The purpose of this randomized controlled experiment is to look into how multistrain probiotic supplementation influences the changes in important biomarkers in people with T2DM. By assessing these indicators, the research aims to shed additional light on the potential of probiotics as a non-pharmacological supplement to traditional T2D therapies, which could lead to more comprehensive and successful T2DM management plans. If successful, multistrain probiotics could be a useful supplement to the current treatment strategies for type 2 diabetes, providing a new, affordable, and accessible way to enhance patient outcomes and lessen the disease’s long-term impacts.

## 2. Methodology

### 2.1. Participants

The study was conducted in a diabetes clinic at SRM Medical College Hospital and Research Centre. The study was registered in the Clinical Trials Registry-India (CTRI/2023/07/055647) after being approved by the institutional ethical committees. The sample size was calculated considering a type I error of 1% and a type II error of 10%, resulting in a required sample size of 61 patients per group. The study examined and enrolled 130 patients with T2DM between August and October 2023. The participants included 79 females and 51 males. A total of 65 participants (22 men and 43 females) were randomly allocated to take a multistrain probiotic supplement, while another 65 (29 men and 36 females) were given a placebo. The participants in the placebo group took vitamin B12 capsules. Samples were collected at two distinct time points, week 0 (baseline, before starting the probiotic or placebo intervention) and week 24 (after the 24-week intervention). Following a thorough explanation of the study, all the participants agreed to sign an informed consent form.

The eligibility criteria for this study were as follows: the participants had to have an established diagnosis of T2DM for at least 6 months prior to the beginning of the study, must not be currently on antibiotics, must be aged between 25 and 70 years, and had to have a HbA1c level of 6.5 or higher. The exclusion criteria included individuals with type 1 diabetes, pregnant women, those with gestational diabetes, and participants who had already begun taking probiotic supplements. The individuals in the probiotic group were instructed to continue their regular eating and exercise routines while taking the supplement twice daily, before meals, in the morning, and at night for 6 months. They were also told not to take any additional probiotics during the study. In order to keep tabs on how many capsules each participant took, compliance was assessed using weekly mobile interviews.

### 2.2. Supplements Characteristics

Fourteen live strains of microorganisms totaling 30 billion colony-forming units (CFUs) per dosage were included in the multistrain probiotic supplement used in the study. Each strain is 2.148 billion CFU per capsule. The strains included in the supplement were *Lactobacillus plantarum* (*L. plantarum*), *L. fermentum*, *L. acidophilus*, *L. casei*, *L. rhamnosus*, *L. reuteri*, *L. salivarius*, *L. paracasei*, *L. gasseri*, *Bifidobacterium bifidum* (*B. bifidum*), *B. lactis*, *B. breve*, *Streptococcus thermophilus*, and *Saccharomyces boulardii.* The participants were instructed to store the capsules away from direct sunlight to preserve viability.

### 2.3. Measurement of Outcomes

Telomere length (TL) was the main outcome, with hs-CRP, HbA1c, and body mass index (BMI) serving as supplementary outcomes. Blood samples were taken after a fasting of 8 to 12 h. A total of 200 µL of whole blood was used for TL and was quantified using the quantitative polymerase chain reaction (qPCR) method. TL compares the sample’s telomere repeat copy number (T) to its single-copy gene copy number (S). The following primer sequences were utilized:

Forward (Telomere): 5′-GGTTTTGAGGGTGAGGGTGAGGGTGAGGGTGAGGGT-3′

Reverse (Teomere): 5′-TCCGACTATCCCTATCCCTATCCCTATCCCTATCCCTA-3′

For 36B4, primers were:

Forward (36B4): 5′-CAGCAAGTGGAAGGTGTAATCC-3′

Reverse (36B4): 5′-CCATTCTATCATCAACGGGTACAA-3′

The procedure used qPCR to compare telomere sequences with 36B4 [13,14]. In total, 5–10 ng of DNA, SYBR Green, and primers for 36B4 and telomeres were included in each reaction (20 µL). Initial denaturation was carried out for 10 min at 95 °C, followed by 25–30 cycles for telomeres at 95 °C for 15 s and 54 °C for 2 min, and 30–35 cycles for 36B4 at 95 °C for 15 s and 58–60 °C for 1 min. The relative telomere length (RTL) was calculated using the 2^−ΔΔCt^ approach. Briefly, the ΔCt for each sample was determined by subtracting the Ct value of the reference gene (36B4) from the Ct value of the telomere region. Next, the ΔΔCt was calculated by subtracting the ΔCt of the control or reference sample from the ΔCt of the sample of interest. Finally, the relative telomere length (T/S ratio) was calculated as 2^−ΔΔCt^, providing a comparison of telomere length relative to the control or reference sample. The hs-CRP levels were determined using immunoturbidometry. High-performance liquid chromatography (HPLC) by D-10, Bio-Rad (Bio-Rad Laboratories, Hercules, CA, USA) was used to determine the levels of HbA1c. Weight (kg)/height (m^2^) was the formula used to determine the body mass index (BMI). We used standardized equipment to measure the participant’s weight and height to ensure accuracy. In order to measure blood pressure, a sphygmomanometer that had been calibrated was used. After a brief five-minute break, the subjects were asked to sit down and take their measurements. To guarantee accuracy, we recorded an average of two readings. A 24 h dietary recall was used to evaluate the participant’s eating habits and nutritional intake, which allowed for an exhaustive examination of their food consumption.

### 2.4. Statistical Analysis

Statistical analysis was carried out using GraphPad Prism version 8. Significant differences between and within groups were identified using a *p*-value of <0.05. Paired sample *t*-tests were used to compare the differences between groups before and after the probiotic treatment. Unpaired *t*-tests were used to determine the differences between the probiotic and placebo groups, allowing for a thorough evaluation of the probiotic’s efficacy. The study utilized both intention-to-treat (ITT) and per-protocol (PP) analyses to evaluate the intervention. The ITT analysis was conducted on the complete set of data, with missing data imputed using the last observation carried forward (LOCF) method. In contrast, the PP analysis was performed on the participants who successfully completed the 24-week intervention and had more than 85% compliance.

## 3. Result

A total of 130 participants were enrolled in the study, with 6 participants (4.6%) withdrawing during the course, leaving 124 participants who completed the 24-week intervention. The final analysis included 62 participants in the placebo group and 62 participants in the test group, all of whom achieved a compliance rate of >85%. Compliance rates were comparable between the two groups, with the placebo group at 91.92% and the probiotic group at 91.98%. For the per-protocol (PP) analysis, all 124 participants who completed the study were included, as they met the compliance threshold, and none consumed additional interventions or external factors, such as other probiotics, that could have confounded the results. Three participants in the multistrain probiotic group were excluded from the experiment. Two of these participants were removed due to non-compliance with the supplementation regimen, while the third participant was excluded because they did not show any response to the treatment. Similarly, three participants in the placebo group were also excluded. Two of these participants were removed for not responding to the placebo, and one participant was excluded due to experiencing stomach issues that interfered with their participation in the study. These exclusions were made to ensure the integrity of the data and to account for any factors that might have confounded the results. Importantly, no major adverse effects were noted among the individuals who consumed the multistrain probiotic supplements throughout the study, demonstrating that T2DM patients tolerated the probiotics well. The comprehensive study investigated the effects of the probiotic intervention on key physiological markers, providing a detailed analysis across probiotic and placebo groups through baseline characteristics, overall group comparisons, and age-stratified results. The baseline characteristics (Table 1) revealed no statistically significant differences between the probiotic and placebo groups, establishing a robust foundation for the subsequent analysis. The participants were well matched across critical parameters, including age (probiotic: 55.0 ± 9.16 years; placebo: 57.6 ± 8.56 years, *p* = 0.111), height (probiotic: 157.2 ± 8.05 cm; placebo: 157.3 ± 8.08 cm, *p* = 0.930), and weight (probiotic: 67.6 ± 10.9 kg; placebo: 70.8 ± 11.67 cm. *p* = 0.092), and initial parameters such as BMI (probiotic: 27.3 ± 4.13; placebo: 28.6 ± 5.04, *p* = 0.127) and glycemic control (HbA1c—probiotic: 7.86 ± 1.61; placebo: 7.51 ± 0.96, *p* = 0.147). Notably, the baseline telomere length showed a slight, non-significant difference between the groups (probiotic: 1.10 ± 0.45; placebo: 0.97 ± 0.37, *p* = 0.092).

The overall group comparison (Table 2) revealed significant within-group and between-group dynamics across multiple physiological markers over the 24-week study period. Telomere length demonstrated a statistically significant reduction in both groups (probiotic: −0.08 ± 0.07, *p* < 0.001; placebo: −0.10 ± 0.07, *p* < 0.001), with a significant between-group difference (*p* = 0.036), suggesting the probiotic intervention may have a modest protective effect on decreased telomere shortening. High-sensitivity C-reactive protein (hs-CRP) showed a more pronounced reduction in the probiotic group (−0.60 ± 0.87, *p* < 0.001) compared to the placebo group (−0.20 ± 0.81, *p* = 0.056), with a statistically significant between-group difference (*p* = 0.010), indicating the potential anti-inflammatory benefits of the probiotic intervention. Glycemic control, measured by HbA1c, demonstrated a significant improvement in the probiotic group (−0.44 ± 1.17, *p* = 0.004) versus a minimal change in the placebo group (−0.05 ± 0.92, *p* = 0.665), with a statistically significant between-group difference (*p* = 0.041).

The age-stratified analysis (Table 3) provided deeper insights into the intervention’s differential effects across age groups. In the younger cohort (30–49 years), the probiotic intervention showed more pronounced benefits. Telomere length decreased from 1.36 ± 0.50 to 1.27 ± 0.48 in the probiotic group (*p* < 0.001) compared to a greater decline in the placebo group from 1.27 ± 0.41 to 1.13 ± 0.382 (*p* < 0.001), with a significant between-group difference (*p* = 0.040). Similarly, the hs-CRP levels demonstrated a significant reduction in the younger probiotic group, dropping from 2.31 ± 0.66 to 1.84 ± 0.71 (*p* = 0.010), while the placebo group showed no significant change (*p* = 0.728). The HbA1c levels in the younger cohort also improved more substantially in the probiotic group, decreasing from 8.34 ± 1.6 to 7.70 ± 0.80 (*p* = 0.037), compared to the placebo group. In contrast, the older cohort (50–69 years) showed more subtle effects. While both groups experienced a reduction in telomere length, the differences were less pronounced, with no statistically significant between-group variation (*p* = 0.320). The hs-CRP and HbA1c changes were similarly less dramatic in this age group, suggesting a potential age-dependent response to the probiotic intervention. The BMI data complemented these findings, with the probiotic group, particularly in the younger cohort, showing a modest but consistent reduction. These multifaceted results suggest that probiotic supplementation may offer benefits across different physiological markers, with the most promising effects observed in younger participants. The study highlights the complex interplay between probiotics, cellular aging, inflammatory responses, and metabolic health, providing valuable insights into the potential nutritional interventions for mitigating age-related physiological changes. While the findings are encouraging, the researchers emphasize the need for further investigation to fully elucidate the mechanisms underlying these observations and to determine the long-term implications of probiotic supplementation across different age groups.

## 4. Discussion

To our knowledge, this is the first study to explore the impact of multistrain probiotics supplementation on telomere length in T2DM. The intricate relationship between telomere dynamics, inflammatory processes, and metabolic health represents a frontier of contemporary medical research, particularly in the context of type 2 diabetes mellitus. Our investigation provides insights into the potential therapeutic role of multistrain probiotics in modulating cellular aging and metabolic parameters.

Telomeres and telomere-associated proteins are essential to the aging process according to the theory of cellular senescence. Age-related metabolic, inflammatory, and neurodegenerative disorders are linked to telomere shortening. Growing data in recent years has demonstrated that people with type 2 diabetes have significantly shorter mean leukocyte telomere lengths than people without diabetes [15,16,17,18,19,20,21]. In line with earlier research, we also discovered that patients with type 2 diabetes had smaller telomere lengths. One widely recognized explanation for the condition in T2DM patients is that elevated oxidative stress brought on by hyperglycemia results in oxidative telomeric DNA damage and shortened telomeres [15]. A study by Shen et al. showed that telomere length in T2DM patients was 0.98, whereas in our study, we observed a slightly increased baseline telomere length of 1.10 in the probiotic group and a similar length of 0.97 in the placebo group [22]. Additionally, Uziel et al. observed that telomere attrition in T2DM was averted by proper glycemic control. The results of our study showed that after taking probiotics for six months, the T2DM patients’ glucose management and telomere length shortening had dramatically improved [23]. Probiotics help regulate blood glucose levels by influencing short-chain fatty acids (SCFAs). SCFAs stimulate L-cells to secrete Glucagon-like peptide-1, which promotes insulin secretion and alleviates T2DM. Additionally, SCFAs reduce the levels of inflammatory markers through intestinal epithelial cells, contributing to lower blood glucose levels [24].

The results of our study showed that using the multistrain probiotic supplements for 24 weeks decreased the serum hs-CRP levels in the T2DM patients when compared to the placebo. In the study by Raygan et al. [25], the mean decrease in hs-CRP levels was −0.8 mg/L, whereas our study observed a mean decrease of −0.6 mg/L. Toejing et al. demonstrated that supplementation with *Lactobacillus paracasei* for 12 weeks in individuals with T2DM led to a reduction in both hs-CRP levels and HbA1c [26]. Probiotic’s impact on serum hs-CRP has been documented among immunocompromised patients [27], pregnant women [28], and patients with rheumatoid arthritis [29]. Consuming a mixture of *L. casei*, *B. breve*, and prebiotic galacto oligosaccharides [30] as *B. longum* [31] has been found to decrease serum hs-CRP levels in immunocompromised patients.

Consuming probiotic yogurt containing *L. acidophilus* and *B. animalis* after 9 weeks of pregnancy [28], administering *Bifidobacteria* supplementation to patients undergoing colorectal cancer resection [32], and administering probiotic bacteria supplementation to patients with stage 3 and stage 4 chronic kidney disease after 6 months [33] have all been linked to similar results.

The serum hs-CRP levels in our study showed a tendency to decline after taking multistrain probiotic supplements. According to a meta-analysis of twelve RCTs, probiotics could reduce HbA1c by 0.54% [34]. On the other hand, our research found that HbA1c decreased by 0.44%, indicating that multistrain probiotics had an impact on glycemic management. The intricacy of the cellular aging pathways is shown by age-specific differences in telomere length responses. The younger age group’s more noticeable results point to possible age-dependent cellular plasticity and probiotic intervention responsiveness. This result emphasizes how crucial customized strategies are for nutritional and therapeutic interventions.

The limitations of the current study include the relatively short intervention period and the need for more comprehensive mechanistic investigations. Future research should explore long-term telomere dynamics, investigate specific molecular mechanisms, and potentially stratify interventions based on age, metabolic status, and individual microbiome compositions.

The study’s findings contribute to the growing body of evidence supporting the potential therapeutic role of probiotics in managing type 2 diabetes. By demonstrating impacts on telomere length, inflammatory markers, and HbA1c, this research opens new avenues for understanding the intricate relationships between cellular aging and metabolic health. In conclusion, the multistrain probiotic intervention demonstrated promising potential in modulating cellular aging markers and metabolic parameters in patients with type 2 diabetes mellitus. The age-specific responses, reduced inflammatory markers, and improved glycemic control suggest a multifaceted therapeutic approach that extends beyond traditional diabetes management strategies.

## Figures and Tables

**Table 1:** Baseline characteristics of participants.

	Probiotic Group	Placebo Group	*p* ^a^
Age	55.0 ± 9.16	57.6 ± 8.56	0.111
Weight, kg	67.6 ± 10.9	70.8 ± 11.67	0.092
Height, cm	157.2 ± 8.05	157.3 ± 8.08	0.930
BMI	27.3 ± 4.13	28.6 ± 5.04	0.127
Systole, mmHg	127.3 ± 17.45	130.2 ± 19.03	0.390
Diastole, mmHg	79.5 ± 9.06	79.7 ± 9.29	0.993
Telomere length	1.10 ± 0.45	0.97 ± 0.37	0.092
hs-CRP mg/L	2.39 ± 0.67	2.44 ± 0.85	0.761
HbA1c %	7.86 ± 1.61	7.51 ± 0.96	0.147

Data are represented as mean ± SD. ^a^ obtained from an unpaired *t*-test. cm = centimeter; kg = kilogram; mmHg = millimeters of mercury; mg/dL = milligrams per liter. BMI = Body Mass Index; hs-CRP = high-sensitivity C-reactive protein; HbA1c = glycated hemoglobin.

**Table 2:** Within-group and between-group comparison.

Probiotic Group					Placebo Group				
	0th Week	24th Week	Change	*p* ^a^	0th Week	24th Week	Change	*p* ^a^	*p* ^b^
TL	1.10 ± 0.45	1.02 ± 0.43	−0.08 ± 0.07	<0.001 *	0.97 ± 0.37	0.86 ± 0.34	−0.10 ± 0.07	<0.001 *	0.036 *
hs-CRP mg/L	2.39 ± 0.67	1.79 ± 0.74	−0.60 ± 0.87	<0.001 *	2.44 ± 0.85	2.23 ± 0.74	−0.20 ± 0.81	0.056	0.010 *
HbA1c %	7.86 ± 1.61	7.42 ± 0.88	−0.44 ± 1.17	0.004 *	7.51 ± 0.96	7.4 ± 1.06	−0.05 ± 0.92	0.665	0.041 *
BMI	27.38 ± 4.13	26.72 ± 4.04	−0.65 ± 1.21	<0.001 *	28.66 ± 5.04	28.52 ± 5.09	−0.13 ± 2.13	0.880	0.104

Data are represented as mean ± SD. ^a^ obtained from a paired *t*-test for the within-group comparisons. ^b^ obtained from an unpaired *t*-test for the between-group comparisons. Statistically significant values are denoted as “*”. TL—relative telomere length; BMI = Body Mass Index; hs-CRP = high-sensitivity C-reactive protein; HbA1c = glycated hemoglobin.

**Table 3:** Age-wise comparison within groups and between groups.

Age	Probiotic Group					Placebo Group				
		0th Week	24th Week	Change	*p* ^a^	0th Week	24th Week	Change	*p* ^a^	*p* ^b^
30–49 (n1 = 25, n2 = 23)	TL	1.36 ± 0.50	1.27 ± 0.48	−0.08 ± 0.08	<0.001 *	1.27 ± 0.41	1.13 ± 0.382	−0.13 ± 0.07	<0.001 *	0.040 *
	hs-CRP mg/L	2.31 ± 0.66	1.84 ± 0.71	−0.46 ± 0.82	0.010 *	2.12 ± 0.78	2.17 ± 0.77	0.05 ± 0.67	0.728	0.024 *
	HbA1c %	8.34 ± 1.6	7.70 ± 0.80	−0.63 ± 1.41	0.037 *	7.73 ± 1.03	7.70 ± 1.13	−0.03 ± 1.14	0.9015	0.115
	BMI	26.94 ± 3.40	26.49 ± 3.13	−0.44 ± 1.23	0.087	27.33 ± 3.65	27.54 ± 3.75	0.20 ± 1.43	0.5102	0.106
50–69 (n1 = 37, n2 = 39)	TL	0.93 ± 0.32	0.85 ± 0.29	−0.07 ± 0.05	<0.001 *	0.80 ± 0.20	0.70 ± 0.17	−0.09 ± 0.06	<0.001 *	0.320
	hs-CRP mg/L	2.45 ± 0.66	1.76 ± 0.76	−0.69 ± 0.90	<0.001 *	2.62 ± 0.84	2.27 ± 0.72	−0.35 ± 0.84	0.014	0.095
	HbA1c %	7.54 ± 1.48	7.22 ± 0.88	−0.32 ± 0.96	0.053	7.39 ± 0.89	7.32 ± 0.99	−0.06 ± 0.77	0.614	0.212
	BMI	27.67 ± 4.54	26.88 ± 4.56	−0.79 ± 1.18	0.003 *	29.45 ± 5.56	29.10 ± 5.66	−0.34 ± 2.42	0.393	0.311

Data are represented as mean ± SD. ^a^ obtained from a paired *t*-test for the within-group comparisons. ^b^ obtained from an unpaired *t*-test for the between-group comparisons. Statistically significant values are denoted as “*”. TL—relative telomere length; BMI = Body Mass Index; hs-CRP = high-sensitivity C-reactive protein; HbA1c = glycated hemoglobin.

## Data Availability

The data presented in this study are available on request from the corresponding author upon reasonable request.
